# Pesticides and Neurologic Symptoms: Kamel et al. Respond

**Published:** 2005-12

**Authors:** Freya Kamel, Beth C. Gladen, Jane A. Hoppin, Dale P. Sandler, Lawrence S. Engel, Michael C.R. Alavanja

**Affiliations:** National Institute of Environmental Health Sciences, National Institutes of Health, Department of Health and Human Services, Research Triangle Park, North Carolina, E-mail: kamel@niehs.nih.gov; Memorial Sloan-Kettering Cancer Center, New York, New York; National Cancer Institute, National Institutes of Health, Department of Health and Human Services, Bethesda, Maryland

Burns and Goldstein raise several issues regarding our paper (Kamel et al. 2005), in which we reported that applicators chronically exposed to moderate levels of pesticide experience more neurologic symptoms. They assert that our measures of exposure and effect are not “meaningful.” We disagree.

Burns and Goldstein state that “‘multiple symptoms’ is not a definable disease or illness.” Although this is true, symptoms cause many medical visits and so are significant to public health. Further, we made no claim that applicators reporting more symptoms had a particular disease. Indeed, in some of our analyses we purposely excluded individuals reporting neurologic disease in order to evaluate associations of pesticide use specifically with symptoms. We studied a mixed group of symptoms, all sometimes associated with neurologic dysfunction or disease, although with varying specificity. Excluding two relatively nonspecific symptoms (headache and fatigue) did not appreciably change the distribution of the symptom variable. The assertion that we limited our analysis to ”a single episode” is inaccurate: our main analyses evaluated multiple rather than single symptoms, and we took symptom frequency into account in our analysis of individual symptoms (Kamel et al. 2005; Table 4). We acknowledged the limitations of cross-sectional analysis in our article. However, the associations we observed were with cumulative pesticide use; accounting for recent use did not change results.

The issue is not whether the symptoms we studied are diagnostic of neurologic or other disease, but whether experiencing these symptoms is associated with pesticide exposure. Burns and Goldstein cite Lundberg et al. (1997) but omit Lundberg et al.’s conclusion that the exposure-related relationship of symptom reporting to organic solvent exposure makes this approach useful for comparing groups with different exposures. At least 23 previous studies used symptom reporting to evaluate neurologic effects of pesticide exposure, with 19 reporting positive associations (Kamel and Hoppin 2004). We extended this approach to a very large group of applicators who had detailed exposure information available.

Burns and Goldstein discuss potential factors related to symptoms, citing Spurgeon’s biopsychosocial model (Spurgeon et al. 1996). We agree that personal and social factors likely influence both experience and reporting of symptoms. However, Spurgeon (2002) noted that

Discussion of the determinants of symptom reporting does not constitute a dismissal of the farmer’s illness but simply a recognition that it is likely to result from a complex interaction of physical, psychological, and social processes.

She described a study of farmers whose symptoms were associated with five factors, one being handling sheep within 48 hr of pesticide dipping. Thus, pesticide exposure may still be associated with increased symptoms even (or perhaps especially) when psychosocial factors are taken into account. Most of the factors Burns and Goldstein list are unlikely to be related to exposure in licensed applicators and so cannot explain the associations seen. Further, confounding by psychosocial factors would likely produce associations with all types of pesticides, but our findings were specific to insecticides. Finally, we do not understand Burn’s and Goldstein’s comment that our findings are “the result … of a common ailment such as influenza”; are they suggesting that pesticide exposure is associated with increased risk of flu?

Burns and Goldstein call our exposure measures limited, citing biomonitoring studies which show that variations in internal exposure are not completely correlated with external exposure. This point is largely irrelevant because the associations seen depend not on identifying the absolute level of pesticide exposure but rather on ranking applicators as relatively more or less exposed. Variation in the degree to which self-reported days of use represents internal exposure is probably nondifferential with respect to symptom reporting, with resulting misclassification likely to bias associations towards the null; the true relationship may be stronger than we observed. Our findings of associations with insecticides only, and with cumulative but not recent exposure, suggest that recall bias does not fully account for our results. We see no problem in combining pesticides for a class-wide analysis, particularly because many grouped pesticides exert effects through similar or related biologic mechanisms. Using class-wide analyses may minimize confounding because most applicators used multiple pesticides. Ultimately, it will be interesting to evaluate the effects of individual chemicals; we are planning such studies.

Thus, our measures of both exposure and effect are sufficient for their purpose, which is to examine the association of symptom reporting with moderate insecticide exposure. Our study clearly demonstrates such an association. Importantly, it is independent of both recent exposure and a history of high exposure or poisoning, suggesting that lifetime exposure at moderate levels may have health consequences. This finding has implications for farmers’ health and deserves to be reported and evaluated further.
